# Methylmalonic acidemia/propionic acidemia – the biochemical presentation and comparing the outcome between liver transplantation versus non-liver transplantation groups

**DOI:** 10.1186/s13023-019-1045-1

**Published:** 2019-04-02

**Authors:** Tzu-Hung Chu, Yin-Hsiu Chien, Hsiang-Yu Lin, Hsuan-Chieh Liao, Huey-Jane Ho, Chih-Jou Lai, Chuan-Chi Chiang, Niang-Cheng Lin, Chia-Feng Yang, Wuh-Liang Hwu, Ni-Chung Lee, Shuan-Pei Lin, Chin-Su Liu, Rey-Heng Hu, Ming-Chih Ho, Dau-Ming Niu

**Affiliations:** 1 Division of Genetics and Metabolism, Department of Pediatrics, Taipei Veterans General Hospital, Taipei, Taiwan; 2 Department of Medical Genetics, National Taiwan University Hospital, Taipei, Taiwan; 3 Department of Pediatrics, MacKay Memorial Hospital, Taipei, Taiwan; 4 Mackay Junior College of Medicine, Nursing and Management, Taipei, Taiwan; 5 Newborn Screening Center, The Chinese Foundation of Health, Taipei, Taiwan; 6 Section of Newborn screening, Taipei Institute of Pathology, Taipei, Taiwan; 7 Division of Rehabilitation, Department of Medical Affairs, Taipei Municipal Gan-Dau Hospital (Managed by Taipei Veterans General Hospital), Taipei, Taiwan; 8 Division of Pediatric Surgery, Department of Surgery, Taipei Veterans General, Taipei, Taiwan; 9 Institute of Environmental And Occupational Health Sciences, Taipei, Taiwan; 10 Department of Surgery, National Taiwan University Hospital, Taipei, Taiwan; 11 Institute of Clinical Medicine, National Yang-Ming University, Taipei, Taiwan; 12 The Medical Science & Techonology Building, (Room 8055) No. 201, Sec.2, Shih-Pai Road, Taipei, Taiwan, Republic of China; 13 Taiwan Medican Mission in Eswatini, Taipei, Taiwan, Republic of China

**Keywords:** Methylmalonic acidemia, Propionic acidemia, Newborn screening, Liver transplantation

## Abstract

**Background:**

Most patients with isolated methylmalonic acidemia (MMA) /propionic acidemia (PA) presenting during the neonatal period with acute metabolic distress are at risk for death and significant neurodevelopmental disability. The nationwide newborn screening for MMA/PA has been in place in Taiwan from January, 2000 and data was collected until December, 2016.

**Results:**

During the study period, 3,155,263 newborns were screened. The overall incidence of MMA mutase type cases was 1/121,356 (*n* = 26), 1 *cobalamin B* was detected and that for PA cases (*n* = 4) was 1/788,816. The time of referral is 8.8 days for MMA patients, and 7.5 days for PA patients. The MMA mutase type patients have higher AST, ALT, and NH_3_ values as well as a lower pH value (*p* < 0.05). The mean age for liver transplantation (LT) is 402 days (range from 0.6–6.7 yr) with 16 out of 20 cases (80.0%) using living donors. The mean admission length shortened from 90.6 days/year (pre-LT) to 5.3 days/year (at 3rd year post-LT) (*p* < 0.0005). Similarly, the tube feeding ratio decreased from 67.8 to 0.50% (*p* < 0.00005). The anxiety level of the caregiver was reduced from 33.4 to 27.2 after LT (*p* = 0.001) and the DQ/IQ performance of the patients was improved after LT from 50 to 60.1 (*p* = 0.07).

**Conclusion:**

MMA/PA patients with LT do survive and have reduced admission time, reduced tube feeding and the caregiver is less anxious.

## Background

**Fig. 1:**
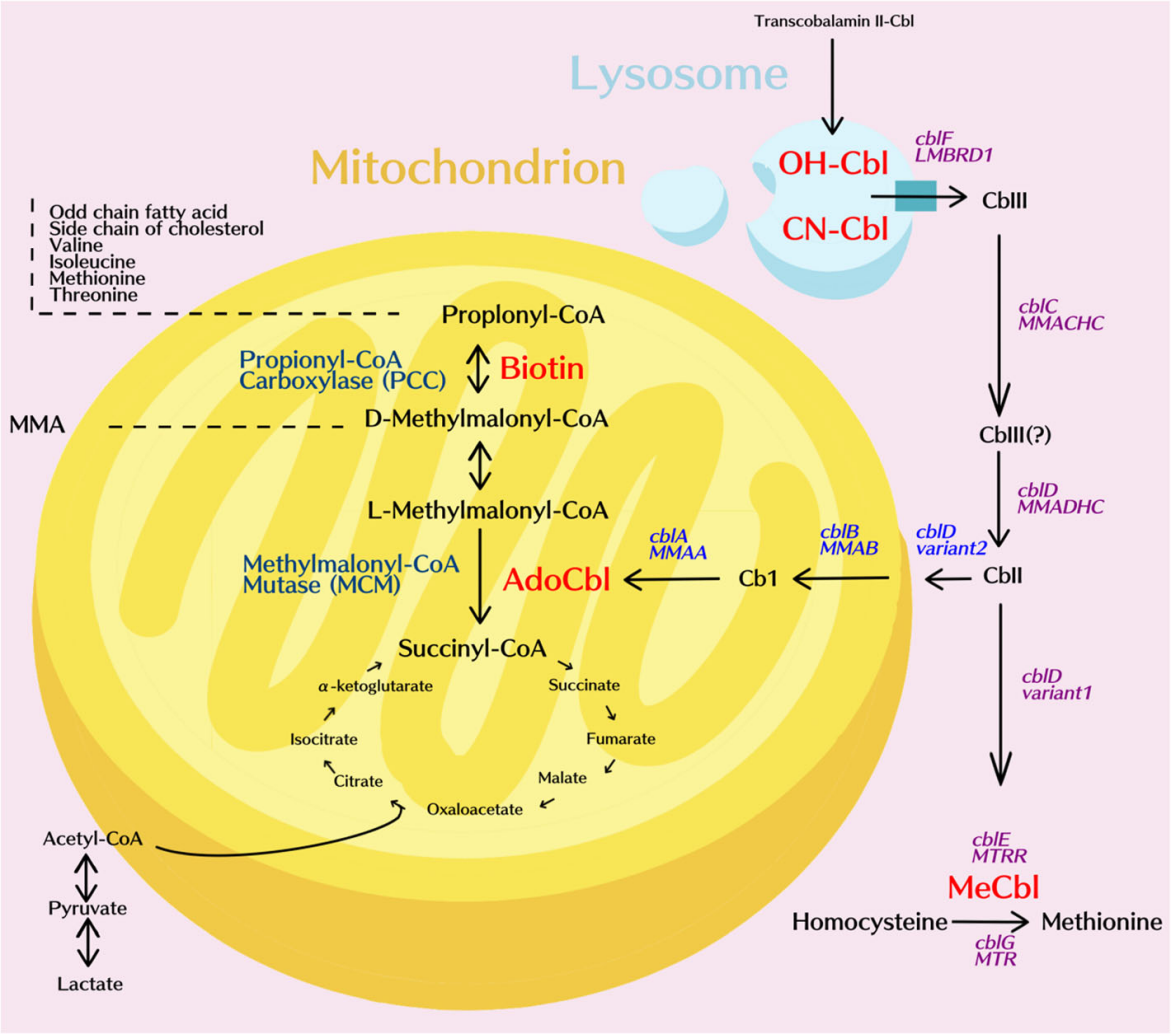
Metabolic pathway of propionyl-CoA and methylmalonyl-CoA. Methylmalonic acidemia (MMA, MIM#251000) is caused either by a defect in methylmalonyl coenzyme A mutase (MUT, encoded by the *mut* gene, on chromosome 6p12.3) or by a defect in the uptake, transport or synthesis of 5’-deoxyadenosylcobalamin (AdoCbl). A disruption of the synthesis of AdoCbl is attributable either to a defect in *cblA* (251100), which is caused by a mutation in the *MMAA* gene located on chromosome 4q31, or to a defect in *cblB* (251110), which is caused by a mutation in the *MMAB* gene located on chromosome 12q24. Combined methylmalonic acidemia and homocystinuria occurs in individuals with mutations in *cblC* (277,400, *MMACHC* gene, located on chromosome 1p34), *cblD* (277,410, *MMADHC* gene, located on chromosome 2q23), and *cblF* (277380). Propionic acidemia (PA MIM#606054, is caused by deficiency in propionyl-CoA carboxylase (PCC), a biotin-dependent carboxylase that is present in the mitochondrial matrix. This enzyme is composed by two subunits, which are encoded by *pccA*, located on chromosome 3q22. 3 and *pccB*, located on chromosome 13q32.3

Methylmalonic acidemia (MMA, MIM#251000) and propionic acidemia (PA, MIM#606054) are autosomal recessive organic acidemias that are characterized by the accumulation of methylmalonate or propionate due to a defect in either methylmalonyl-CoA mutase (MUT) or propionyl-CoA carboxylase (PCC). MMA is able to be further divided into a MUT deficiency or a defect in cobalamin (*cbl*) metabolism (the coenzyme of MUT) that can include uptake (*cblF*), transport (*cblC,D*) or synthesis (*cblA,B*) of functional 5′-deoxyadenosylcobalamin (AdoCbl). Defects in *cblC*, *cblD* and *cblF* induce a combination of methylmalonic aciduria and homocystinuria owing to a concurrent impairment of the synthesis of methylcobalamin (MeCbl). (Fig. 1).

These *mut* mutations are classified into two subgroups: *mut*^*0*^, where there is complete loss of enzyme activity (deficiency) and *mut*^*−*^, where there is residual but insufficient enzyme activity. The majority of *mut* mutations are associated with the *mut*^*0*^ phenotype [1], and these mutations result in significantly higher levels of mortality and morbidity.

Many classical MMA/PA patients deteriorated rapidly while suffering from early poor feeding, lethargy, vomiting, and metabolic acidosis that extent to encephalopathic coma; they then succumb early in infancy. For these classical patients, dietary restriction (a low-protein, high-energy diet) together with oral medication have remained the core therapies for decades [2]. However, despite intensive medical efforts, frequent decompensation still occurs and complications seem to be inevitable. The implement of liver transplantation (LT) for MMA *mut*^*0*^ and PA patients since 1990s has seemed to offer a chance to reduce the number of life-threatening decompensation events [3] as well as other long-term complications and moreover, less dietary restrictions [4–6].

In Taiwan, MMA was officially incorporated into the nationwide newborn screen (NBS) from March, 2000 onwards. Since 2006, it has been a mandatory screening test. There are three national newborn screening centers in Taiwan; these are National Taiwan University Hospital (NTUH), the Taipei Institute of Pathology (TIP) and the Chinese Foundation of Health (CFOH). A previous study of 1,495,132 neonates covering the period March 2000 to June 2009 identified thirteen MMA patients (incidence: 1/101,625) and two PA cases (incidence: 1/660,562) [7]. This early study did not, however, evaluated the management and prognosis of this group of patients to any degree. In this article, our aim was to extend the screening period to December 2016 and to analyze a range of parameters related to the diagnosed children, particularly whether they had or did not have liver transplantation.

## Materials and methods

### Referral criteria

From January, 2000 to December, 2016, neonates with elevated propionylcarnitine (C3) and with an elevated C3/acetylcarnitine (C2) ratio, who were identified at NTUH, TIP and CFOH, were included in this study. The referral criteria of the three units are presented in Table 1. The “borderline” cutoff values were modified during the pilot studies (2000–2006) of our NBS centers at approximately 4 standard deviations above the mean, whereas the “positive” cutoff values were established at about twice the borderline cutoff values [7]. At CFOH and TIP, the borderline cutoff value for C3 was 6μM, while that at NTUH the value was 4.74. The positive cutoff value for the ratio of C3/C2 at TIP/CFOH was either a value greater than or equal to 0.3 or 0.35 or a C3 > =12 or 14, respectively. The infant would be referred immediately to confirmatory medical centers: Taipei Veterans General Hospital... Furthermore, screened newborns with a C3/C2 between 0.25 and 0.3 or 0.35, respectively, or an intermediate C3 level would be requested for a repeat blood sampling sent to TIP/CFOH. Based on the data submitted from TIP and CFOH, from 2004 to 2011, the overall screen-positive rate yielded 77.78%; from 2012 and onward, after methylmalonic acid (mma) used at a second tier, the rate increased to 83.3%. At NTUH, the reference value for resampling is C3/C2 ratio 0.26. Cases with C3 > =8.8 and C3/C2 > =0.26 will be referred immediately to NTUH. Cases with abnormal C3 or C3/C2 will then be determined by mma level to see if he/ she is likely to have MMA (mma > =1μM) or PA (<1μM).

**Table 1:** Referral criteria of three NBS centers in Taiwan

	CFOH borderline/positive	TIP borderline/positive	NTUH borderline/positive
C3 (μM)	6/14	6/12	4.74/8.8 (1st tier)
C3/C2	0.25/0.35	0.25/0.3	0.26
mma (μM)	4.2 (2nd tier)	Pos/Neg	1 (2nd tier)

The first number indicates the cutoff point for resampling, whereas the second number stands for value of immediate referral to confirmatory medical center: NTUH/Taipei Veterans General Hospital. Note in CFOH and in NTUH, a second tier of mma level would be conducted in case of high C3 and/ or C3/C2 value.In TIP, a quantitative test of mma would be performed after detection of elevated C3 or C3/C2

### Biochemical analysis

The information on all the MMA/PA patients was recorded in detail and this included demographic data, biochemical markers and initial presentation. In addition, to aid early differentiation, we also recruited fourteen false positive cases for better analysis. These were neonates who were referred to our center yet later their tandem mass and urine organic acid turned out to be free of MMA/PA. They were classified as “transient MMA/PA” without verifiable reason (except one case was diagnosed of primary hyperoxaluria from the organic urine analysis result) or can be traced to maternal deficiency of cobalamin. Our aim was, to compare their initial laboratory data with true MMA/PA newborns.

To compare the prognosis of the LT and non-LT groups, we have traced the data from another 5 mutase MMA cases born before the introduction of NBS. Descriptive statistics were calculated for the demographic and clinical characteristics. Because *cblA* patients are mild, B12 responsive and *cblC* carries a different pathophysiology with remethylation dominating the pathophysiology and management, these 2 types of patients were removed from our study cases. The data were classified to *mut* (MUT), *cblB*, and PA patients.

### The parameters for evaluation

The parameters studied included survival rate, DQ/IQ performance, admission length and tube feeding time. These were compared using the unpaired 2-sample *t* test. The quality of life scale from evaluation of patients is shown in Table 2 and values were classified into “good”, “mildly impaired”, “moderately impaired” and “severely impaired” according to the “unscheduled admissions per year”, the “tube feeding ratio” and the “use of metabolism-correcting/immunosuppressive agents”. This approach was modified from a previous report of Morioka et al. [8]. Patients were categorized into the worst group if any of the features matched. The tube feeding ratio is defined as the days of tube feeding (either from nasogastric, orogastric tube or from gastrostromy), collected from the chart records and/or the recall of the main caregiver, then divided by 365*100%. The larger the tube feeding ratio, the less capable the patients could feed by mouth to provide sufficiency energy in avoidance of aggravation of catabolism. Beck Anxiety Inventory (BAI) Chinese Version was utilized to assess the anxiety level of the main caregiver and the questionnaire was distributed in 2015. We request the parent recall the status before LT and at 1 year after LT. The sum of the scores from the questionnaire could range from 21 to 84. Scores between 21~28 were considered “a minimal level of anxiety”, while those that were 29~36 were scored as “mild anxiety”, those that were 37~46 were scored as: “moderate anxiety” and those > 47 were identified as having “severe anxiety” [9].This 21-item scale show high internal consistency (Cronbach’s alpha, α = 0.92) and test-retest reliability (r = 0.75) over 1 week [10].. Cognitive function was assessed using the cognitive subtest of the Bayley-III (0–36 months old), the Wechsler Preschool (36mo-72mo) and the Wechsler IQ IV (>72mo) according to the chronological age of the subject or the functional performance of the subject. Mild cognitive developmental impairment was defined by a score between 55 and 70, while moderate impairment had a range from 40 to 55, severe impairment had a range from 25 to 50 and profound impairment had a score less than 25. The mean follow-up period after LT was 68 months. The perioperative mortality is defined as death occurring within 30 days after LT regardless if in or out of the hospital.

**Table 2:** Classification of quality of life

	Unscheduled admissions/yr	Tube feeding	Metabolism-correcting/ immunosuppressive agents
Good	≦ 2 times	(−)	1
Mildly impaired (if any)	3–5	< 25%	2–3
Moderately impaired (if any)	6–8	25–50%	4–5
Severely impaired (if any)	≧9	> 50%	≧6

We revised from Morioka et al. on the classification of quality of life. Unscheduled admission is defined as any admission due to unexpected acute decompensation, infection…and etc., which was collected from hospital records. The tube feeding ratio counts the days of length of nasogastric, orogastric tube or from gastrostomy within 1 year, then divided by 365*100%, indicating the severity of feeding quality, based on the nursing records and/or the recell of the main caregiver

## Results

During the study period, 27 MMA cases were identified: 26 *mut* type, 1 *cblB* and 4 PA cases. This was out of a total screened population of 3,155,263 newborns. Hence the updated incidence in Taiwan for each of the organic acidopathy are 1/121,356 (*mut*), 1/631,053 (*cbl*) and 1/788,816 (PA).

Gene mutation analysis was carried out on the MUT patients and in total 56 alleles were analyzed. In Taiwan, G427D (*mut*^*0*^) was found to be the most prevalent MMA *mut* mutation (41.1%) and this was followed by L328F (*mut*^*0*^, 10.7%), c. 1677-1G > A (*mut*^*0*^, 7.1%), R152X (*mut*^*0*^, 5.4%), R228X (*mut*^*0*^, 5.4%), and R108H (*mut*^*−*^, 5.4%).

Before the era of NBS, it required on average a total of 37.4 days from birth to reach a confirmed diagnosis for MMA *mut*^*0*^ neonates and this has now been dramatically decreased to only 8.8 days for MMA patients, and 7.5 days for PA patients (*p* < 0.05). This strongly supports and affirms the need for NBS, which allow the quick and early detection of both of these diseases.

In order to explore the acylcarnitine profile of these individuals, each group of patients was compared with each other group that formed the study (Table 3). The false positive patients (9.05 ± 2.74, 4.65~13.70μM) were found to have overlapping C3 values with the *mut* patients (11.21 ± 3.05, 6.30~17.52 μM). However, surprisingly, the false positive group can even show a higher C3 level than the *cblB* patients (6μM). The PA patients tend to have the highest mean C3 level (11.11 ± 2.72, 9.07~15.1μM) and there was a significant gap when they were compared with the *cbl* group. (false>*cbl*; PA > *cbl*).

**Table 3:** Initial acylcarnitine profile among MMA mutase, MMA *cblB*, PA and false positive patients

	False Positive (*n* = 14)	MMA		PA (*n* = 4)
		mut (*n* = 18)	cbl (n = 1)	
C3	9.05 ± 2.74 (4.65~13.70)	11.21 ± 3.05 (6.30~17.52)	**6**	11.11 ± 2.72 (9.07~15.1)
C3:C2	0.38 ± 0.39 (0.10~1.24)	0.80 ± 0.50* (0.36~2.36)	0.43	1.54 ± 1.02^*^ (0.60~2.97)
Average methylmalonic acid	45.25 ± 45.20 (11.69~122.30)	271.16 ± 145.59^*^ (125.8~634)	14.8	0.97^$^

This table showed there is overlapping data among each group. * indicates significant difference (*p* < 0.05) compared to false positive group; $ indicates significant lower compared to mutase type patients. It reveals in terms of initial C3 level, PA/ mut > false; regarding to initial C3: C2, mut/PA > false/cbl; and MMA level: mut > false/cbl > PA

When the C3:C2 ratios were examined, the *mut* (0.80 ± 0.50, 0.36~2.36) and PA (1.54 ± 1.02, 0.60~2.97) patients were found to have significantly higher values than the false positive (0.49 ± 0.42, 0.10~1.24) and *cbl* patients (0.43). (*mut*/PA > false/*cbl*). With such cases it was not possible to separate well the false positive individuals and the cobalamin deficient patients. The *mut* subjects (271.16 ± 145.59, 125.8~634) showed the highest initial MMA level compared to the false positive group (45.25 ± 45.20), the *cbl* group (14.8) and the PA group (0.97). Thus the C3:C2 ratio appears to provide better discrimination when identifying classical MMA/PA patients. One of the false positive patients presented with a C3 of 12.2, a C3:C2 ratio of 1.24 and a MMA level of 122.3μM; it later turned out that this abnormal propionylcarnitine profile was due to a severe maternal B12 deficiency secondary to autoimmune gastritis. Hence, according to our study, a C3:C2 value greater than 1.25 was only observed in the *mut*/PA patients. Furthermore, neonates who possess a MMA level higher than 123 μM have a high risk of MUT deficiency.

The patients’ initial biochemical information upon admission is presented in Table 4**.** The *mut* patients can be seen to have the worst biochemical manifestation of the disease. They have the highest aspartate transaminase (AST), alanine transaminase (ALT), serum creatinine (Cre), ammonia (NH_3_) and serum glycine levels as well as the lowest pH values (*p* < 0.05). The *cbl*/PA patients presented with intermediate values. An elevated ALT (> 40 IU) or Cre (> 0.7 mg/dl) was only observed in the *mut* patients. Only the *mut*/PA patients seem to show an elevated AST (> 45 IU) and/or Bun (> 19 mg/dl) upon initial blood sampling. Notably, the NH_3_ and pH values were within the normal range for the false positive group even though some MMA/PA individuals also showed normal values initially. In the case of hyperammonemia (NH_3_ > 150μg/dl), attention is needed immediately as such a referred case is at high risk. Severe metabolic acidosis (bicarbonate < 13.5 mmol/L) only occurred in the *mut* patients.

**Table 4:** Initial biochemical data of among MMA mutase, MMA *cblB*, PA and false positive patients upon their admission

	False positive (*n* = 10)	MMA		PA (*n* = 4)
		mut (*n* = 18)	cbl (*n* = 1)	
ALT(IU) (0–40)	15.00 ± 4.83 (6–21)	43.64 ± 48.34* (11-166)	14	18.00 ± 9.83^a^ (6–30)
AST(IU) (5–45)	30.5 ± 21.89 (19–92)	98.64 ± 115.96^*^ (19-451)	34	44.50 ± 20.94^a^ (21–69)
Bun(mg/dl) (7–20)	10.67 ± 4.37 (6–18)	13.18 ± 8.88 (2–35)	8	13.08 ± 10.65 (7–29)
Cre(mg/dl) (0.5–1.5)	0.23 ± 0.14 (0.1–0.39)	0.62 ± 0.47^*^ (0.1-2.0)	0.42	0.50 ± 0.16* (0.3-0.69)
NH3(μg/dl) (5–150, newborn)	70.58 ± 27.25 (35.8–132)	388.89 ± 299.07^*^ (92-1244)	253	341.00 ± 227.28^*^ (84-637)
pH value (7.40 ± 0.08)	7.43 ± 0.08 (7.305–7.54)	7.28 ± 0.14^*^ (6.94-7.47)	7.55	7.37 ± 0.11 (7.229–7.476)
HCO3 (20 ± 4)	20.54 ± 3.19 (14.5–26.3)	13.97 ± 6.38 (2.0–25.3)	8.7	18.10 ± 4.07 (13.5–22)
Glycine (μM) (110–240)	227.7 ± 52.61 (190.5–264.9)	533.49 ± 221.21* (238-1163.5)	148.9	592 ± 106.06 (84–667)

^*^indicated significant difference (*p* < 0.05) compared to false positive group

^a^indicated significant difference compared to mutase type patients

According to our institutional policy, MMA/PA patients are eligible for LT once they reach 8 kg, regardless of their actual age. None of the MMA *cbl* type patients received LT, but, by way of contrast, eighteen patients with MMA *mut* patients along with 2 PA cases received LT. The mean age for liver transplantation (LT) is 402 days. Most of these transplants (16/20, 80.0%) received a living donor transplant from a matched relative. The rest received a cadaveric liver. After LT, an abrupt decline in the C3/C2 ratio was obvious (− 32.41% in the LT group versus + 34.80% in the non LT group: *p* = 0.04). Their serum MMA levels also dropped by 79.99% (from 373.20 ± 1004.09μM to 97.63 ± 63.19μM). Among the patients in the non LT group, the MMA levels also dropped, specifically from 146.93 ± 433.87 to 109.39 ± 116.76; this decrease of 17.68% being attributable entirely to medical control of the condition (as shown in Table. 5). Interestingly, both groups revealed an elevation for unknown reasons in C3 levels among the affected children as they grew older, and interestingly the LT-group showing a greater increase (+ 371.07% compared to + 241.55% for the non-LT group). The increase of C3 may reflect the liberation of diet control and gradual deterioration of kidney function. We followed up 9 MMA patients postoperatively and discovered 3/9 (30%) entered CKD stage III at mean age of 6.2 (5.1–6.8).

**Table 5:** Acylcarnitine profile of LT group: pre-LT and post-LT and of non-LT group

LT(+)	Pre-LT	Post-LT	%	*p*
C3	14.41 ± 14.55	22.17 ± 8.71	+ 371.07%	0.05
C3:C2	2.046 ± 1.5673	0.9335 ± 0.3807	−32.41%	0.01^*^
MMA	373.20 ± 1004.09	97.63 ± 63.19	−79.99%	0.15
LT(−)	Initial	Latest	%	*p* compared with LT group
C3	11.18 ± 4.27	51.23 ± 51.07	+ 241.55%	0.12
C3:C2	0.8148 ± 0.27	1.1 ± 0.3920	+ 34.80%	*0.04
MMA	146.93 ± 433.87	109.39 ± 116.76	−17.68%	0.20

*indicated significant difference compared to false positive group (*p* < 0.05)

In LT(+) group: Pre-LT acylcarnitine level was collected 1 month before LT. Post-LT level was recruited at 4.1 ± 0.5 year after LT. In LT(−) group: Initial level indicates value at 1 month and latest values indicates at 3.9 ± 0.7 year

**Fig. 2:**
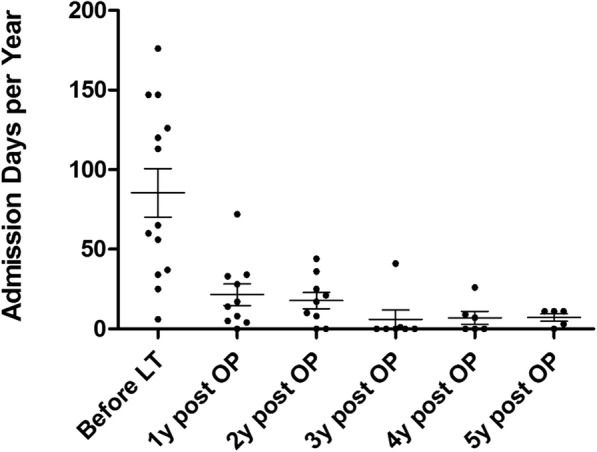
Admission length times of MMA and PA patients before and after LT

**Fig. 3:**
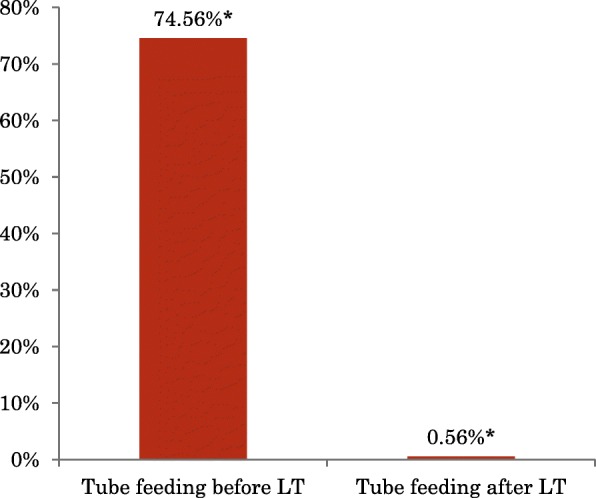
Tube feeding time of MMA mut patients, displayed in percentage, before and in the 2nd year after LT

The annual admission length (days per year) for all LT patients decreased from 90.6 ± 52.8 days (1 year before LT) to 28.2 ± 20.4 days (1st year post-LT) and then to 18.4 ± 14.4 days (2nd year post-LT), 5.3 ± 14.3 days (3rd year post-LT), 7.7 ± 9.2days (4th year post-LT) and 6.2 ± 4.7 days (5th year post LT). Thus there was a significant reduction in admission days between before and after LT (*p* < 0.0005) (Fig. 2) and it was noticeable that admission length remained relatively stable after 2 years post-transplant. The tube feeding ratio also decreased dramatically from 67.8% before LT to 0.50% (nine *mut* patients and one PA patients, *p* < 0.0002) in the 2nd year after LT among the MMA *mut*/PA patients (Fig. 3). Many parents reported that the patient was almost always fed by mouth within 6 months of transplantation. Patients with a gastrostomy all underwent a gastrostomy closure operation within 252 to 881 postoperative days.

The caregivers of MMA and PA patients also were found to be less anxious after LT. The Beck Anxiety Inventory Chinese Version scores of these individuals dropped from 33.4 to 27.2 (*p* = 0.001, *n* = 10), which implies a move from “mild anxiety” to “a minimal level of anxiety”. For the one PA patient, the score declined from 44 to 35 (n = 1), as shown in Table 6.

**Table 6:** Beck Anxiety Inventory (Chinese Version) as an objective evaluation of anxiety from the main caregiver, before and at 1 year after LT

	Pre-LT	Post-LT	*P*
MMA (10 cases)	33.4	27.2	0.001
PA (1 case)	44	35	NA

**Fig. 4:**
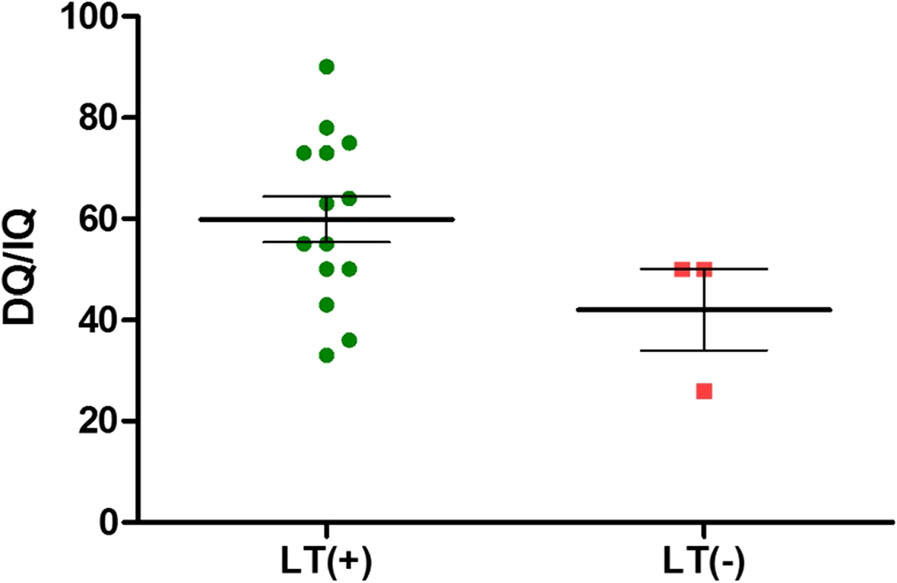
Functional assessment (DQ/IQ) of isolated MMA and PA patients, categorized in LT(+) and LT(−) group

In terms of functional assessment, which includes the development quotient and the intelligence quotient, MMA *mut* patients with LT were found to have a better prognosis (mean = 60.13) than those without LT (50), although this was not statistically significant (*p* = 0.07). Here we have excluded one late-onset *mut*^*−*^case. This female patient developed her first episode of metabolic acidosis at the age of 4 years [11]. Her DQ/IQ was assessed to be 113. Another case had undergone a profound neonatal intraventricular hemorrhage that had severe neurological sequelae and this case was also excluded. The *cblB* patients had values of 100. Both post-LT PA patients were scored to be 64. The overall analysis of isolated MMA and PA cases are illustrated as in Table 7 and as in scattered plot in Fig. 4.

**Table 7:** Functional assessment (DQ/IQ) of isolated MMA and PA patients, according to diagnosis before/after the initiation of NBS; and whether or not received LT

	Before era of NBS	After era of NBS	Overall
LT (+)	59.7 ± 15.3 (43–73, *n* = 3)	59.9 ± 17.9 (33–90, *n* = 11)	59.9 ± 16.8 (n = 14)
LT(−)	26	50 (50,50)	42 (n = 3)

Seventeen patients had at least 1 DQ/IQ assessment. In this table, isolated MMA (12 patients) and 2 PA patients were analyzed as a whole. Before era of NBS, 3 MMA cases underwent LT and they were assessed at age 11 ± 3.6 (yr). In contrast, 9 MMA and 2 PA patients underwent LT after the introduction of NBS. They were assessed at age 5.9 ± 3.6 (yr). Only 3 MMA (including 1 before NBS) did not receive LT. Two of them received DQ/IQ assessment at 9.5 and 10.2 (yr). The only one MMA LT(−) case born before NBS received DQ/IQ test at 3.6 yr. and expired 1 month afterwards. The DQ/IQ difference of LT(+)/LT(−) does not reach significance (*p* = 0.11)

The perioperative mortality of the MMA *mut* patients with LT stands at 5.6% (1/18), and the 5-year mortality rate of LT in Taiwan for MMA patients is 16.7%, which contrasts with the situation for patients without LT, where the mortality stands at 25% (2/8). One MMA case s/p LT expired on postoperative day 9 due to a pulmonary hemorrhage. A second case expired at 3.5 months due to a post-LT infection and one case died accidentally at 3 years and 3 months after LT due to suffocation during sleep. Two *mut* cases without LT died due to acute exacerbation of the disease. Both post-LT PA cases have survived to date, while one PA case without LT died due to acute exacerbation of the disease.

Brain MRI was also performed on the patients. The findings included focal or diffuse hypomyelination and T2 hyperintensity. One 7-years old MMA *mut*^*0*^ boy who had undergone LT at the age of 8 months developed an infarct in the bilateral globi pallidi. No optic neuropathy was detected among the MMA/PA patients with or with LT born after the introduction of NBS**.**

## Discussion

Although LT cannot cure classical genetic MMA/PA, many experts are now convinced that transplantation should be performed for the sake of providing an improved quality of life for the patient [8], as well as better growth and improved metabolic control [12]. However, in order to obtain a better prognosis, some centers have suggested that LT be performed within the first year of life [13]. Even though the mortality of mutase type patients has decreased from 60% in the 1980s; to 40% in the 2000s [14, 15] and recent studies have indicated that the survival rate at the end of first year can be as high as 85 to 92% in the United States [16], even though the management of these diseases remains very difficult. LT should be considered to be only a partially curative treatment. In Japan, MMA and PA rank number 5 and 6 in the list of metabolic disorders requiring pediatric LT [15]. In the United States, out of 5672 pediatric LT cases that were carried out from 2002 to 2012, there were twenty seven MMA cases, of which ten underwent LT and 17 underwent a combined liver-kidney transplant when the patients were renal insufficient [17]. As collective experience with LT has accumulated, the boundary between LT as a standard life-saving procedure and LT as a rather aggressive life-improving therapy has becoming reduced [15]. Biochemically, according to a previous study, post-LT serum MMA levels are decreased to 13.8% ± 9.2% (range 1.25–26.1%) of their preoperative levels by LT [3]and the results obtained by our team are consistent with this (20.01%). Furthermore, plasma MMA have been found to be reduced to 3% of their pre-dialysis level in cases where there has been a combined liver-kidney transplantation [18]. In spite of the decreased C3/C2 ratio, we still noticed that there was a noticeable increase in C3 concentration after LTThis could be explained by gradual loosening of protein restriction, persistent chronic deterioration of renal function and increased skeletal muscle mass since the skeletal muscle is recognized to produce a large amount of metabolite in mma [19].

In 2016, a Japanese group carried out a similar survey of MMA patients and it was concluded LT benefits MMA patients in terms of cognitive performance [20] and we have found a similar trend with our patients.

In conjunction with prophylaxis to treat the acute decompensation episodes, the improved quality of life among our MMA and PA patients after LT was confirmed as resulting in less anxiety among their parents. Financially, Li et al. simulated the costs and outcomes of LT among MMA/PA patients and the results indicated that the LT group was remarkably more cost-effective compared to the simple nutrition support group [16]. This was associated with an improvement in the patient’s quality of life due to a dramatic decrease in the hospitalization time needed to recover from metabolic decompensation and the relaxation in dietary restriction. In addition to the above, for PA patients, LT has been found to reverse dilated cardiomyopathy and long QT syndrome of 4 cases after more than 10 years of follow-up [21, 22].

In the literature it has been suggested that several other factors may affect the long-term prognosis of MMA and PA patients and these include cobalamin responsiveness, the duration of coma and peak level of NH_3_. MMA *mut*^*−*^ individuals seem to have a better outcome than *mut*^*0*^ individuals [23]. In addition, we have included in our study *cblB* and *mut* patients born before the year 2000 and these individuals may have received suboptimal treatment initially. Therefore, we speculate that the IQ outcome for our patients may not be entirely correlated with their cobalamin defect type.

The genotypes of Taiwanese patients appear to differ from the People’s Republic of China, where the most common defect (12 out of 78 mutant alleles, 15%) is c.729_730insTT (p.D244Lfs*39) [24], which only occurred once out of 56 mutants alleles (1.8%) from our data in Taiwan. On the contrary, the genotypes in Taiwan appear similar to Southern part of China, where the leading mutant allele is G427D, followed by c.1630_1631GG > TA (p.G544X) [25] Our findings also differ from those of Japan, where the most frequent mutations are p.E117X, c.385 + 5G > A, p.R369H, p.L494X, and p.R727X [26]. We also found that 17 (81%) patients were classified as *mut*^*0*^. This percentage exceeds the European frequency of 58% [1].

Peri-operative and post-operative care are crucial for MMA and PA patients who undergo LT. Patients should be monitored meticulously in intensive care units regarding bleeding control, acid-base/electrolyte balance, infection and compromised respiration. In out center, moreover, they are also scheduled to annual bile drainage revision and liver biopsy to detect any signs of occlusion or rejection. They may become relatively immunocompromised after discharge and opportunistic infection is always a risk. It has been suggested that the chronic use of immunosuppressants is likely to increase the risk of hypertension, anemia, malignancy, bone disease, cardiovascular diseases, renal dysfunction and psychiatric disorders [16]. Our institute showed 5 MMA and 1 PA patients with metabolic disease after LT were eventually weaned off tacrolimus, and fiveremained tacrolimus-free for more than 2 years.. Pediatric patients are immunologically naïve thus render tacrolimus withdrawal likely in selected recipients (histologically stable graft function > 1 year if transplant at < 1 year of age, or stable up to 2 years if transplant at > 1 year of age) [27]. Two *mut* patients are prescribed sodium bicarbonate due to renal tubular acidosis that is secondary to their MMA, and three *mut* patients are prescribed an antihyperuricemic agent to control elevated uric acid that might be induced by the immunosuppressant [28] or secondary to methylmalonate deposition as an injury to renal tubule cells [29] The study in Lucile Packard Children’s Hospital at Stanford reported 6 from 14 patients MMA underwent LT and 8 patients received a combined liver-kidney transplantation (CLKT) with 100% survival rate at a mean follow-up of 3.25 ± 4.2 years [30]. All of them underwent a deceased donor transplantation. To compare the patients’ demographic data with ours, the age to receive transplantation apparently much older (mean age 8.2 years, range 0.8–20.7) than our patient group (0.6–6.7). There was only one patient among Stanford group under age of 1 year to whom LT was performed. For infant of inborn error of metabolic disease, we have gained plentiful experience from the management. Since 2004, we have conducted in our center more than 30 LTs. Besides MMA/PA, we also did LT for patients with urea cycle disorders, maple syrup urine disease, Wilson disease, homozygous familial hypercholesterolemia and homocysteinuria. Due to scarcity of renal transplant, it is less feasible to carry out CLKT in Taiwan.

Several reports have shown that there is likelihood that the functioning of other organs of both MMA *mut* and PA patients may deteriorate, including the central nervous system (especially the presence of basal ganglia lesions, with more than 56% of PA patients and 36% of MMA patients having such lesions in one survey [31]), the heart (predominantly in PA patients), the presence of optic neuropathy (MMA > PA), the pancreas and the kidneys (MMA). Some patients may still eventually require dialysis and kidney transplantation independent of LT to alleviate MMA-related end-stage renal disease. Patients with *mut*^*−*^ or cblB deficiency may not present milder symptoms [2].

Before LT, many patients did not have a chance of surviving beyond infancy; nevertheless, after the LT, they still do face a life that involves struggle. In such circumstance care should always be taken to avoid long-term extrahepatic complications and any such complications should be treated in a timely manner. For MMA and PA patients, decades of follow-up and the establishment of a registry of associative parameters are necessary. The joint participation of multi-specialists is indispensable to further investigations of MMA and PA patients and these need to include metabolism physicians, pediatric hepatologists, the pediatric transplantation team, metabolic nutritionists and the nursing staff. We intend to continue to detect new MMA and PA cases at the earliest possible opportunity and we will provide them with our au-courant care of quality.

In the future, gene therapy may shed some light on the cure of these inborn errors. Chandler et al. have previously developed in 2008 murine models with limited effective treatment by direct hepatic injection of an adenovirus that expressed the MUT gene. Later in 2010, recombinant adeno-associated viruses with were proved by the same team to successfully treat 26/27 *Mut*^−/−^ neonatal mice via one-time hepatic administration, while prolonging their survival beyond 1 year (except 1 demise following a blood collecting procedure) [32] Besides, it is also reported that messenger RNA (mRNA) encoding mutase encapsulated into lipid nanoparticles for single intravenous injection resulted in 75–80% reduction of plasma mma in mice. Direct mRNA treatment other than gene delivery could allegedly avoid immune response and mutagenesis risks because it does not transit to the nucleus [33].

The limitations of this study contain relatively smaller patients sample size and sustain to non-randomized clinical trial. Whether or not to receive LT depends on joint discussion with informed consent from the family.

## Conclusion

NBS using acylcarnitine profile including C3, C3:C2 and serum methylmalonate concentration drastically shortened the time to diagnosis. MMA/PA patients with LT possess improved biochemical data, survival and quality of life: less admissions, reduced tube feeding and less anxiety to the caregiver. Further follow-up for long-term complication is still indicated.

## Abbreviations

AdoCbl: 5’-deoxyadenosylcobalamin
ALT: Alanine transaminase
AST: Aspartate transaminase
BAI: Beck Anxiety Inventory
C2: Acetylcarnitine
C3: Propionylcarnitine
cbl: Cobalamin
CFOH: Chinese Foundation of Health
CLKT: Combined Liver-Kidney Transplantation
Cre: Serum creatinine
DQ/IQ: Developmental quotient/intelligence quotient
LT: Liver transplantation
MeCbl: Methylcobalamin
mma: Methylmalonic acid
MMA: Methylmalonic acidemia
MUT/mut: Methylmalonyl-CoA mutase
NBS: Newborn screen
NH3: Ammonia
NTUH: National Taiwan University Hospital
PA: Propionic acidemia
TIP: Taipei Institute of Pathology
